# Risk factors for morbidity in both donor and recipient following minimally invasive donor hepatectomy: a systematic review

**DOI:** 10.1097/JS9.0000000000003524

**Published:** 2025-10-09

**Authors:** Sang-Hoon Kim, Ki-Hun Kim, Surendran Sudhindran, Dieter C. Broering

**Affiliations:** a Division of Liver Transplantation and Hepatobiliary Surgery, Department of Surgery, Asan Medical Center, University of Ulsan College of Medicine, Seoul, Korea; b Department of Gastrointestinal Surgery and Solid Organ Transplantation, Amrita Institute of Medical Sciences, Kochi, India; c Organ Transplant Center of Excellence, King Faisal Specialist Hospital and Research Centre, Riyadh, Saudi Arabia

**Keywords:** living-donor liver transplantation, minimally invasive donor hepatectomy, morbidity, risk factor, systematic review

## Abstract

**Background:**

Minimally invasive donor hepatectomy (MIDH), including laparoscopic, laparoscopy-assisted, and robotic donor hepatectomy, is an evolving technique in living donor liver transplantation, but its technical complexity presents potential risks for both donors and recipients. This study purposes to systematically review and identify key risk factors for donor and recipient morbidity after MIDH.

**Methods:**

A systematic search of electronic databases was performed to identify studies published between January 2001 and December 2024 that reported significant risk factors for donor and recipient complications after MIDH. Risk factors for overall or major complications, biliary complications (bile leak or biliary stricture), or open conversion were summarized using odds ratios or hazard ratios with 95% confidence intervals derived from multivariate analysis.

**Results:**

In total, eight studies reported significant risk factors for donor or recipient after MIDH. Risk factors for donor complications included unfavorable anatomical characteristics (short hepatic ducts, multiple hepatic ducts/arteries/veins, and large graft) and operative factors (increased operative time and blood loss). Conversion from laparoscopic to open was related to high BMI. Recipient risk factors included biliary variations, portal vein thrombosis, hepaticojejunostomy, prolonged operative time, massive transfusion, and high Model for End-Stage Liver Disease scores. Robotic surgery was linked to favorable donor and recipient outcomes.

**Conclusion:**

Risk factors for donor and recipient morbidity after MIDH include anatomical, operative, procedural, donor, and recipient factors. Notably, biliary variation of graft is key contributor for both donor and recipient morbidity. Given the limited studies on risk factors, multicenter studies with larger sample sizes are essential to validate these findings.

## Introduction

Minimally invasive donor hepatectomy (MIDH), including laparoscopic donor hepatectomy (LDH), laparoscopy-assisted donor hepatectomy (LADH), and robotic donor hepatectomy (RDH), has emerged as a transformative approach in living-donor liver transplantation (LDLT)^[1–5]^. This evolution reflects the growing importance of reducing donor morbidity while maintaining optimal graft outcomes^[6]^. Although MIDH offers significant advantages such as decreased postoperative pain, faster recovery, and improved cosmetic satisfaction compared with traditional open donor hepatectomy (ODH), it remains a technically demanding procedure with inherent risks for both donors and recipients^[7–11]^. These risks include intraoperative bleeding, biliary complications, and the potential need for conversion to open surgery, which can adversely affect clinical outcomes^[1]^. Understanding the implications of these risks is essential for improving safety and optimizing the clinical results of these minimally invasive approaches^[12,13]^. A clear understanding of the risk factors influencing morbidity, graft quality, and recipient outcomes is essential in improving safety and optimizing the clinical results of these minimally invasive approaches^[13–19]^.

**HIGHLIGHTS**

- Key risk factors for donor and recipient morbidity in MIDH were identified.
- Robotic donor hepatectomy is feasible for both donor and recipient morbidity.
- Biliary variation in the graft is a key contributor to donor and recipient morbidity.

Previous studies on donor and recipient outcomes following MIDH have provided valuable insights but are often limited by small sample sizes, heterogeneity in surgical techniques, and insufficient statistical power in risk factor identification^[1,3,15–17]^. Although multivariate analyses have been applied in several studies, the lack of comprehensive comparisons across cohorts undergoing LDH, LADH, and RDH leaves gaps in understanding regarding the predictors of morbidity^[20–27]^. This systematic review aims to comprehensively evaluate current evidence on risk factors identified through multivariate analysis – focusing on donor and recipient morbidities – to guide future practice and research, propose risk reduction strategies, and develop more effective MIDH protocols, ultimately improving the safety and efficacy of MIDH in LDLT.

## Methods

### Data collection and search strategy

This systematic review followed the guidelines outlined in the Cochrane Handbook for Systematic Reviews of Interventions^[28]^, Preferred Reporting Items for Systematic Reviews and Meta-Analyses (PRISMA)^[29]^, and Assessing the Methodological Quality of Systematic Reviews 2 (AMSTAR 2)^[30]^. As this study was entirely based on published data, institutional review board approval or patient consent was not required. This study was also reported in accordance with the Transparency in the Reporting of Artificial Intelligence (TITAN) criteria (Supplemental Digital Content, available at http://links.lww.com/JS9/F300)^[31]^.

A comprehensive literature search was performed across multiple databases, including Embase, Cochrane Library, Web of Science, and PubMed, including publications from January 2001 to November 2024. Detailed information on the search strategy and criteria is provided in Table 1. The study protocol was registered in the Research Registry under the Unique Identifying Number review registry1929.

**Table 1:** Literature search strategies


Database	Search strategies
Embase	#5 #3 AND #4
01/01/2000–31/10/2024	#4 #1 OR #2
	#3 (donor AND hepatectomy OR donor) AND liver AND resection AND [2000-2024]/py
(*N* = 300)	
	#2 (robot OR “robot assisted surgery”) AND [2000-2024]/py
	#1 (laparoscopic OR “laparoscopic assisted” OR (laparoscopic AND assisted)) AND [2000-2024]/py
Web of Science	#5 #3 AND #4
01/01/2000–31/10/2024	#4 #1 OR #2
	#3 (ALL=(donor hepatectomy)) OR ALL=(donor liver resection)
(*N* = 517)	#2 (ALL=(robot)) OR ALL=(robot assisted)
	#1 ALL=(laparoscopic) OR ALL=(laparoscopic assisted)
Cochrane Library	#5 #4 AND #3
01/01/2000–31/10/2024	#4 #1 OR #2
	#3 (donor hepatectomy):ti,ab,kw OR (donor liver resection):ti,ab,kw
(*N* = 32)	
	#2 (Robot):ti,ab,kw OR (robot assisted):ti,ab,kw
	#1 (laparoscopic):ti,ab,kw OR (laparoscopic assisted):ti,ab,kw
PubMed	#5 (#4) AND (#3) Filters: from 2000/1/1 to 2024/10/31
01/01/2000–31/10/2024	#4 (#1) OR (#2) Filters: from 2000/1/1 to 2024/10/31
	#3 (donor hepatectomy) OR (donor liver resection) Filters: from 2000/1/1 to 2024/10/31
(*N* = 424)	
	#2 (robot) OR (robot assisted) Filters: from 2000/1/1 to 2024/10/31
	#1 (laparoscopic) OR (laparoscopic assisted) Filters: from 2000/1/1 to 2024/10/31

### Study selection

Two investigators independently extracted data from each included study using a standardized data extraction form. To ensure consistency and minimize errors, the extraction form was pilot-tested on a subset of studies prior to full data extraction. Any discrepancies between the two reviewers were first addressed through discussion to reach consensus. If disagreements persisted, a third investigator was consulted to resolve the discrepancies. The inclusion criteria were defined based on the PICOS framework as follows: (1) P (Participants): living liver donors who underwent donor hepatectomy and their recipients; (2) I (Intervention): studies analyzing multivariate risk factors for postoperative complications in donors or recipients following LDLT; (3) C (Comparator): ODH was used as a reference to assess the safety and efficacy of MIDH, including LDH or RDH; (4) O (Outcomes): risk factors for postoperative complications identified through multivariate analysis in cohorts in which MIDH was performed, reported as odds ratios or hazard ratios (HRs) with 95% confidence intervals (CIs); and (5) S (Study design): full-text, original articles published in English, including retrospective single-arm or comparative studies involving MIDH cohorts. The exclusion criteria were as follows: (1) studies lacking multivariate risk factor analysis for postoperative complications following donor hepatectomy through MIDH; and (2) non-original studies such as case reports, letters, abstracts, expert opinions, editorials, reviews, or meta-analyses.

### Data extraction

Two independent researchers extracted data from the included studies. The extracted information comprised the publication year, country, study design, study period, sample size, patient demographics (age and sex), and outcomes of multivariate analyses related to complications in both donors and recipients.

## Results

### Literature search

**Figure 1.**
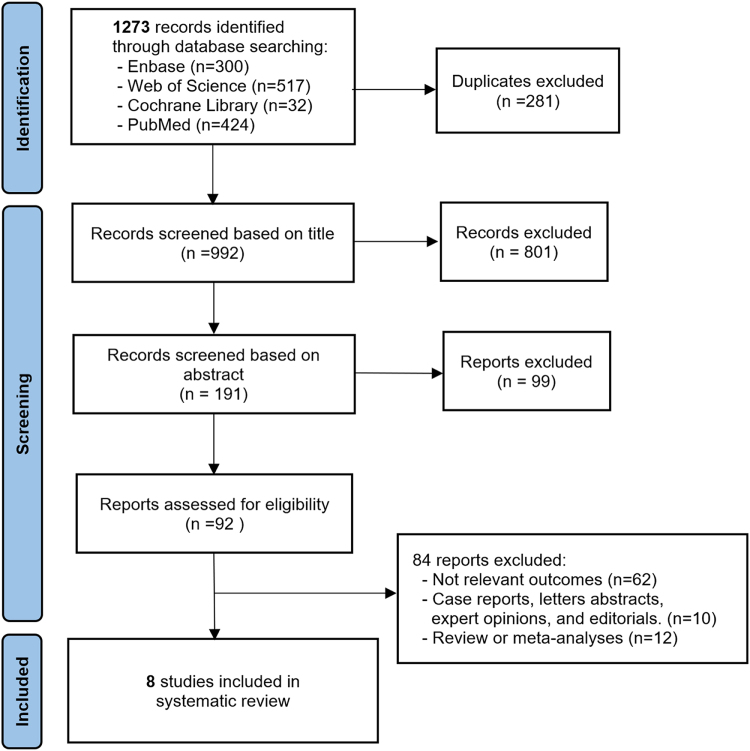
PRISMA flowchart of study inclusion.

A total of 1273 studies were initially identified from electronic database searches. After removing duplicates and conducting a title and abstract screening, 191 articles were identified as potentially eligible for further evaluation. Of these, 84 articles were excluded due to the following reasons: reporting irrelevant outcomes, non-original full-text articles, reviews, or meta-analyses. Finally, eight studies^[20–27]^ met the inclusion criteria and were included in this systematic review. The study selection process is illustrated in the PRISMA flowchart (Fig. 1).

### Study characteristics

The baseline characteristics of the included studies are summarized in Table 2. This systematic review comprised eight studies,^[20–27]^ including two single-arm^[24,25]^ and six comparative studies^[20–23,26,27]^ published from 2019 to 2024.

**Table 2:** Study characteristics and complications of donor and recipients

Study/Year	Country	Study arm	*N*	Donor complication						Recipient complication				
	Design/period			Overall Cx	Major Cx	BC	BL	BS	OC	Overall Cx	Major Cx	BC	BL	BS
Park *et al* 2019^[20]^	Korea	ODH	197	–	13 (6.6)	8 (4.0)	8 (4.0)	0	–	–	81 (41.1)	–	–	–
	RM/2013–2017	LDH	91	–	14 (15.4)	12 (13.2)	11 (12.1)	1 (1.1)	5 (5.5)	–	42 (46.2)	–	–	–
Rhu *et al* 2021^[21]^	Korea	LDH	144	18 (24.5)	6 (8.1)	–	–	–	–	75 (52.1)	50 (34.7)	65 (45.1)	25 (17.4)	40 (27.7)
	R/2016–2019													
Rhu *et al* 2022^[22]^	Korea	ODH	269	46 (17.1)	14 (5.2)	–	–	–	–	186 (69.1)	120 (44.6)	148 (55)	64 (23.8)	65 (12.8)
	R/2013–2021	LDH	506	82 (16.2)	34 (6.7)	–	–	–	–	296 (58.5)	188 (37.2)	229 (45.2)	84 (31.2)	164 (32.4)
Soubrane *et al* 2022^[23]^	France	LADH	237	65 (27.4)	18 (7.6)	20 (8.4)	–	–	6 (2.5)	–	–	–	–	–
	R/2007–2017	LDH	175	43 (24.6)	20 (11.5)	15 (8.6)	–	–	11 (6.4)	–	–	–	–	–
Rhu *et al* 2023^[24]^	Korea	LDH	636	107 (16.8)	40 (6.2)	31 (4.9)	21 (3.3)	10 (1.6)	10 (1.6)	–	–	–	–	–
	R/2013–2022													
Kim *et al* 2023^[25]^	Korea	LDH	543	50 (9.2)	24 (4.4)	19 (3.5)	16 (2.9)	3 (0.6)	9 (1.7)	–	–	–	–	–
	R/2010–2018													
Raptis *et al* 2024^[27]^	Saudi Arabia	ODH	414	–	–	–	–	–	–	251 (61)	128 (31)	63 (15)	51 (12.3)	20 (4.8)
	R/2011–2023	RDH	425	–	–	–	–	–	–	178 (42)	90 (21)	35 (8)	23 (5.4)	10 (2.4)
Raptis *et al* 2024^[26]^	Saudi Arabia	ODH**^a^**	646	106 (16)	5 (0.8)	–	–	–	–	–	–	–	–	–
	R/2011–2023	LDH**^a^**	165	13 (8)	0	–	–	–	6 (3.6)	–	–	–	–	–
		RDH**^a^**	913	35 (4)	1 (0.1)	–	–	–	2 (0.2)	–	–	–	–	–
		ODH**^b^**	193	–	–	–	–	–	–	128 (66)	37 (19)	6 (3.1)	–	–
		LDH**^b^**	129	–	–	–	–	–	–	92 (71)	32 (25)	9 (7.0)	–	–
		RDH**^b^**	341	–	–	–	–	–	–	172 (50)	50 (15)	6 (1.8)	–	–
		ODH**^c^**	453	–	–	–	–	–	–	274 (61)	140 (31)	66 (15)	–	–
		LDH**^c^**	36	–	–	–	–	–	–	24 (67)	16 (44)	6 (17)	–	–
		RDH**^c^**	572	–	–	–	–	–	–	243 (43)	131 (23)	46 (8)	–	–

BC, biliary complication; BL, bile leakage; BS, biliary stricture; LADH, laparoscopic-assisted donor hepatectomy; LDH, laparoscopic donor hepatectomy;*N*, number; OC, open conversion; ODH; open donor hepatectomy; RDH, robotic donor hepatectomy; RM, retrospective-matched; R, retrospective.

^a^ indicates donor hepatectomy for all recipient

^b^ indicates donor hepatectomy for pediatric recipients

^c^ indicates donor hepatectomy for adult recipients.

### Risk factors for donor complications

#### Overall and major donor complications

Four studies^[20,23,25,26]^ reported multivariate analysis results for overall donor complications in cohorts in which MIDH was performed, with three studies^[22,23,25]^ also addressing major complications Table 3. Raptis *et al* identified robotic donor surgery (HR, 0.46; 95% CI, 0.33–0.63; *P* < 0.001), use of a left lateral graft (HR, 0.41; 95% CI, 0.25–0.66; *P* < 0.001), and a donor age ≥35 years (HR, 0.55; 95% CI, 0.31–0.93; *P* = 0.035) as favorable factors for overall complications in a cohort (*N* = 1724) of ODH (*n* = 646), LDH (*n* = 165), and RDH (*n* = 913)^[26]^. In an LDH cohort (*N* = 91), Park *et al* reported the following significant risk factors for overall complications: a right hepatic duct of <1 cm (HR, 4.05; 95% CI, 1.11–14.88; *P* = 0.035) and multiple hepatic veins (HR, 9.86; 95% CI, 1.32–73.53; *P* = 0.026)^[20]^. Moreover, in an international multicenter cohort (*N* = 412) of LAHD (*n* = 237) and LDH (*n* = 175), Soubrane *et al* identified open conversion (HR, 2.914; 95% CI, 1.004–8.463; *P* = 0.035) and estimated blood loss (EBL) (HR, 1.919; 95% CI, 1.218–3.055; *P* = 0.005) as significant risk factors for overall complications, while major complications were significantly related to open conversion (HR, 2.797; 95% CI, 1.006–7.779; *P* = 0.049) and operative time (HR, 1.665; 95% CI, 1.055–2.627; *P* = 0.028)^[23]^. Notably, in a single-center cohort of ODH (*n* = 269) and LDH (*n* = 506), Rhu *et al* found no significant risk factors for major complications related to graft weight, graft type, anatomic variations, or study period^[22]^. In contrast, in a Korean multicenter cohort undergoing right lobectomy (RL)-LDH (*n* = 543) conducted by Kim *et al*, a graft weight of ≥700 g (HR, 2.66; 95% CI, 1.31–5.41; *P* = 0.007), EBL of ≥385 mL (HR, 4.84; 95% CI, 2.50–9.38; *P* < 0.001), and operative time of ≥400 min (HR, 2.46; 95% CI, 1.25–4.88; *P* = 0.01) were significant risk factors for overall complications, while major complications were significantly associated with a graft weight of ≥700 g (HR, 4.01; 95% CI, 1.67–9.62; *P* = 0.002) and operative time of ≥400 min (HR, 3.84; 95% CI, 1.60–9.21; *P* = 0.003)^[25]^.

**Table 3:** Risk factors for donors and recipients identified through multivariate analysis


Study	Cohort	Complication type	Risk factors	Estimates (95% CI)	*P*
Donor risk factors					
Park *et al* 2019^[20]^	LDH for RL (*N* = 91)	Overall complication	Right hepatic duct <1 cm	4.05 (1.11–14.88)	0.035
			Multiple hepatic vein	9.86 (1.32–73.53)	0.026
Soubrane *et al* 2022^[23]^	LDH/LADH for LL and RL (*N* = 412)	Overall complication	Open conversion	2.91* (1.004–8.46)	0.049
			EBL (mL)	1.93* (1.22–3.06)	0.005
		Major complication	Open conversion	2.80* (1.01–7.78)	0.049
			Surgery duration	1.67* (1.06–2.63)	0.028
Rhu *et al* 2022^[22]^	ODH/LDH for RL, LL, LLS, RPS, LTS, and Segment II (*N* = 775)	Major complication	No variables are identified as risk factors	–	–
Rhu *et al* 2023^[24]^	LDH for RL, LL, LLS, RPS, LTS, and Segment II (*N* = 636)	Bile leakage	Division-free margin <5 mm from main duct	2.62 (1.03–6.69)	0.043
			Two hepatic arteries	13.85 (4.09–46.79)	<0.001
			EBL (mL)	1.002 (1.000–1.003)	0.008
			Pringle maneuver	0.30 (0.11–0.82)	0.018
		Biliary stricture	Bile leakage	11.90 (2.77–51.08)	0.001
	LDH for RL (*N* = 586)	Bile leakage	Two hepatic arteries	18.58 (4.76–72.54)	<0.001
			Pringle maneuver	0.26 (0.09–0.73)	0.011
			Estimated blood loss	1.002 (1.000–1.003)	0.016
Kim *et al* 2023^[25]^	LDH for RL (*N* = 543)	Overall complication	Graft weight ≥700 g	2.66 (1.31–5.41)	0.007
			EBL ≥385 mL	4.84 (2.50–9.38)	<0.001
			Operation time ≥400 min	2.46 (1.25–4.88)	0.01
		Major complication	Graft weight ≥700 g	4.01 (1.67–9.62)	0.002
			Operation time ≥400 min	3.84 (1.60–9.21)	0.003
		Biliary complication	Graft weight ≥700 g	4.34 (1.40–13.45)	0.01
			Operation time ≥400 min	4.16 (1.34–12.88)	0.01
		Open Conversion	BMI ≥ 30 kg/m^2^	22.72 (3.53–146.39)	0.001
Raptis *et al* 2024^[26]^	ODH/LDH/RDH for RL, LL and LLS (*N* = 1724)	Overall complication	Robotic donor surgery	0.23 (0.15–0.34)	<0.001
			Left lateral section graft	0.41 (0.25–0.66)	<0.001
			Donor age ≥ 35 y	0.55 (0.31–0.93)	0.035
Recipient risk factors					
Rhu *et al* 2021^[21]^	LDH for RL (*N* = 144)	Bile leakage	Multiple bile duct in graft	4.25 (1.66–10.90)	0.003
		Biliary stricture-free survival	Non-type I bile duct	2.21* (1.14–4.29)	0.018
			Hepaticojejunostomy	3.36* (1.09–10.35)	0.035
			Cut and clip method	0.46 (0.22–0.96)	0.038
Rhu *et al* 2022^[22]^	ODH/LDH for RL, LL, LLS, RPS, LTS, and Segment II (*N* = 775)	Major complication	Portal vein thrombosis Grade 4	5.49 (1.49–20.20)	0.001
Raptis *et al* 2024^[27]^	ODH/RDH for RL (*N* = 839)	Overall complication	RBC transfusion >500 mL	1.58 (1.10–2.27)	0.013
			MELD Na >25	2.26 (1.45–3.57)	<0.001
			Robotic donor surgery	0.46 (0.33–0.63)	<0.001
			Male recipient	0.61 (0.43–0.86)	0.005
			Single duct graft	0.71 (0.51–0.99)	0.042
Raptis *et al* 2024^[26]^	ODH/LDH/RDH for RL, LL, and LLS (*N* = 663)	Overall complication in pediatric recipients	Operation time >6 hr	1.59 (1.05–2.45)	0.032
			RBC transfusion >250 mL	1.86 (1.31–2.67)	0.001
			Robotic donor surgery	0.51 (0.34–0.75)	0.001
	ODH/LDH/RDH for RL, LL, and LLS (*N* = 1061)	Overall complication in adult recipients	Operation time >6 hr	1.45 (1.08–1.95)	0.015
			RBC transfusion >500 mL	1.46 (1.09–1.96)	0.012
			MELD-Na >25	1.57 (1.10–2.25)	0.013
			Acute liver failure	3.07 (1.19–9.51)	0.031
			Robotic donor surgery	0.48 (0.36–0.63)	<0.001
			Male recipient	0.66 (0.50–0.87)	0.004


BMI, body mass index; CI, confidence interval; EBL, estimated blood loss; LADH, laparoscopic-assisted donor hepatectomy; LDH, laparoscopic donor hepatectomy; LL, left lobe; LLS, left lateral segments; LTS, left trisection; MELD, Model for End-stage Liver Disease; ODH, open donor hepatectomy; RBC, red blood cell; RDH, robotic donor hepatectomy; RL, right lobe; RPS, right posterior section.

Estimates are odds ratios

^*^ indicates hazard ratios.

### Donor biliary complications

Two single-arm studies^[24,25]^ on LDH reported multivariate analysis results for donor biliary complications (Table 3). In a cohort of cases (*N* = 636) undergoing RL-, left lobectomy (LL)-, and left lateral sectionectomy (LLS)-LDH, Rhu *et al* identified a division-free margin of <5 mm from the main duct (HR, 2.624; 95% CI, 1.030–6.686; *P* = 0.043), the presence of two hepatic arteries in the graft (HR, 13.836; 95% CI, 4.092–46.789; *P* < 0.001), and EBL (HR, 1.002; 95% CI, 1.000–1.003; *P* = 0.008) as significant risk factors for bile leakage, while the Pringle maneuver was a favorable factor (HR, 0.300; 95% CI, 0.110–0.817; *P* = 0.018)^[24]^. In addition, biliary stricture was significantly associated with bile leakage (HR, 11.902; 95% CI, 2.773–51.083; *P* = 0.018). In the RL-LDH cohort (*n* = 586), significant risk factors for bile leakage included two hepatic arteries in the graft (HR, 18.576; 95% CI, 4.757–72.542; *P* < 0.001) and EBL (HR, 1.002; 95% CI, 1.000–1.003; *P* = 0.016), while the Pringle maneuver remained a favorable factor (HR, 0.258; 95% CI, 0.091–0.732; *P* = 0.011)^[24]^. In another study, Kim *et al* reported a graft weight of ≥700 g (HR, 4.01; 95% CI, 1.67–9.62; *P* = 0.002) and operative time of ≥400 min (HR, 3.84; 95% CI, 1.60–9.21; *P* = 0.003) as significant risk factors for biliary complications in a Korean multicenter RL-LDH cohort (*n* = 543)^[25]^.

### Open conversion

One study^[25]^ reported multivariate analysis results for open conversion in LDH (Table 3). In the RL-LDH cohort (*n* = 543), a body mass index (BMI) of ≥30 kg/m^2^ (HR, 22.72; 95% CI, 3.53–146.39; *P* = 0.001) was identified as a significant risk factor for open conversion. Currently, no studies have reported the risk factors for open conversion in RDH^[25]^.

### Risk factors for recipient complications

#### Overall and major recipient complications

Two studies^[26,27]^ from the same group in Saudi Arabia identified key risk factors associated with overall recipient complications (Table 3). Raptis *et al* analyzed a cohort (*N* = 839) that underwent RL-ODH (*n* = 414) and RL-RDH (*n* = 425) and identified a red blood cell (RBC) transfusion of >500 mL (HR, 1.58; 95% CI, 1.10–2.27; *P* = 0.013) and Model for End-Stage Liver Disease (MELD)-Na score of >25 (HR, 2.26; 95% CI, 1.45–3.57; *P* < 0.001) as significant risk factors for overall recipient complications, while robotic donor surgery (HR, 0.46; 95% CI, 0.33–0.63; *P* < 0.001), a male sex (HR, 0.61; 95% CI, 0.43–0.86; *P* = 0.005), and single-duct grafts (HR, 0.71; 95% CI, 0.51–0.99; *P* = 0.042) were identified as favorable prognostic factors^[27]^. In an adult recipient cohort (*N* = 1061) of patients who underwent RL-, LL-, and LLS-ODH (*n* = 453), -LDH (*n* = 36), and -RDH (*n* = 572), the significant risk factors for overall complications were identified as an operative time of >6 hours (HR, 1.45; 95% CI, 1.08–1.95; *P* = 0.015), RBC transfusion of >500 mL (HR, 1.46; 95% CI, 1.09–1.96; *P* = 0.012), and MELD-Na score of >25 (HR, 1.57; 95% CI, 1.10–2.25; *P* = 0.013), while favorable factors included robotic donor surgery (HR, 0.48; 95% CI, 0.36–0.63; *P* < 0.001) and a male sex (HR, 0.66; 95% CI, 0.50–0.87; *P* = 0.004)^[26]^. Moreover, in a pediatric recipient cohort (*N* = 1061) of patients who underwent RL-, LL-, and LLS-ODH (*n* = 193), -LDH (*n* = 1239), and -RDH (*n* = 341), an operative time >6 hours (HR, 1.59; 95% CI, 1.05–2.45; *P* = 0.032) and RBC transfusion of >250 mL (HR, 1.86; 95% CI, 1.31–2.67; *P* = 0.001) were significant risk factors for overall complications, while robotic donor surgery was a favorable factor (HR, 0.51; 95% CI, 0.34–0.75; *P* = 0.001)^[26]^. Furthermore, in a Korean single-center cohort (*N* = 775) comprising patients who underwent ODH (*n* = 269) and LDH (*n* = 506), grade 4 preoperative portal vein thrombosis in recipients was a key risk factor for recipient major complications (HR, 5.49; 95% CI, 1.49–20.20; *P* = 0.001)^[22]^.

### Recipient biliary complications

One single-arm study^[21]^ on LDH reported multivariate analysis results for recipient biliary complications (Table 3). Rhu *et al* analyzed an adult recipient cohort (*N* = 144) of patients who underwent RL-LDH and identified the presence of multiple bile ducts in the graft as a significant risk factor for bile leakage (HR, 4.252; 95% CI, 1.659–10.900; *P* = 0.003); additionally, biliary stricture-free survival was significantly associated with bile duct types other than type 1 (HR, 2.214; 95% CI, 1.143–4.289; *P* = 0.018)^[21]^.

## Discussion

The disparity between the demand for liver transplantation and the availability of suitable living donors remains a significant challenge in LDLT^[32]^. To overcome this issue, many transplant centers have adopted extended donor selection criteria to expand the living donor pool, considering various factors such as anatomic variation, low remnant liver volume, low graft-to-recipient weight ratio, and old donor age^[32–38]^. However, ensuring the safety of both donors and recipients must remain the highest priority. Notably, MIDH – including laparoscopic and robotic approaches – is currently gaining widespread acceptance. Although LDH is well established for minor procedures such as LLS, evidence supporting its safety in major donor hepatectomy remains limited, with similarly insufficient data available for RDH^[5]^. Therefore, the adoption of MIDH requires a thorough evaluation of significant risk factors during the donor selection process to ensure the safety of both donors and recipients. This careful approach is essential in ensuring that the use of advanced minimally invasive surgical techniques does not compromise clinical outcomes^[32]^.

Thus far, seven studies have identified various risk factors for postoperative complications in living liver donors^[20–26]^. A recent comparative study of RDH, LDH, and ODH found that robotic procedures, LLS, and a donor age of ≥35 years were favorable prognostic factors, highlighting the safety of robotic platforms for donor surgery^[26]^. In contrast, in LDH cohorts, anatomical factors such as a short right hepatic duct (<1 cm) and multiple hepatic veins were found to be significant risk factors for overall complications^[20]^. Additionally, an international multicenter study comparing LDH and LADH identified open conversion, an increased EBL, and a prolonged operative time as critical risk factors for donor morbidity^[23]^, while a Korean multicenter study on LDH for right liver donation identified a graft weight of ≥700 g, EBL of ≥385 mL, and operative time of ≥400 minutes as key predictors of donor complications. Regarding donor biliary complications in an LDH cohort, bile leakage was associated with division-free margins of <5 mm from the main duct, two hepatic arteries, and increased EBL, while biliary stricture was strongly related to bile leakage^[24]^. Interestingly, the Pringle maneuver was identified as a protective factor, likely due to its role in minimizing blood loss and reducing operative time^[24]^. Another study identified a graft weight of ≥700 g and operative time of ≥400 minutes as significant risk factors for donor biliary complications^[25]^. These findings emphasize that a large graft weight, unfavorable anatomical features (short bile ducts, multiple hepatic veins/arteries), and operative factors (blood loss and prolonged surgical time) are significant predictors of donor morbidity. Therefore, to address these challenges, careful preoperative donor evaluation is essential. The Pringle maneuver and robotic platforms may reduce biliary complications and improve donor safety, especially in donors with favorable anatomy.

The risk of conversion from MIDH to open surgery is a critical concern in liver transplantation, with conversion rates varying depending on the type of procedure and institutional expertise. In non-MIDH settings, conversion rates range from 5.4% for LLS to 6.25% for minimally invasive right-sided hepatectomy^[39,40]^, with robotic and laparoscopic procedures showing rates of 1.1% and 2.2%, respectively^[41]^. These rates are influenced by factors such as hypertension, RL, laparoscopic techniques, and challenging dissections due to inflammation or adhesions^[42]^. Conversion is associated with increased morbidity, including bile leaks, infections, and perioperative bleeding, as well as longer operative times, increased blood loss, and extended hospital stays^[43,44]^.

The reported conversion rates for LDH range from 0.0% to 7.4%, reflecting variability across studies and centers^[25,41,44–46]^. Major causes of open conversion during LDH have been reported to include portal vein injury, portal vein thrombosis, uncontrolled bleeding, remnant bile duct injury, difficult hilar dissection, large graft size, and small remnant graft volume^[20,23,25]^. Among these, the most common and significant cause is portal vein injury^[20,23,25]^. Previously, a multicenter study documented a conversion rate of 1.1% in RDH^[25]^; in contrast, a recent single-center series including 913 RDH cases reported an exceptionally low conversion rate of 0.2%^[26,27]^. Factors influencing the likelihood of conversion include anatomical variations, donor obesity, the surgeon’s position on the learning curve, and cumulative surgical experience^[25,39,40,44]^. Notably, only one study identified a BMI ≥30 kg/m^2^ as a significant risk factor for open conversion from MIDH in an LDH cohort^[25]^. Therefore, the analysis of risk factors associated with conversion from MIDH should be incorporated into future research.

In total, four previous studies investigated the risk factors associated with morbidity and complications in recipients following MIDH. In cohorts undergoing RDH, ODH, or LDH, a prolonged operative time (>6 hours), massive transfusion (>500 mL), and high MELD-Na score (>25) were significant predictors of overall recipient complications, while favorable prognostic factors included robotic donor surgery, single-duct grafts, and male donor sex, with robotic donor surgery significantly reducing complications in both adult and pediatric recipients^[26,27]^. However, as these findings are based on single-institution data, further studies are required to validate the feasibility of RDH. In addition, grade 4 portal vein thrombosis was significantly related to major complications in recipients^[22]^. Regarding biliary complications in recipients when using RL, biliary variation with multiple ducts and biliary reconstruction with hepaticojejunostomy were identified as key risk factors, while bile duct division using the cut-and-clip method was a favorable predictive factor^[21]^. These findings highlight the multifactorial nature of recipient complications, which are influenced by recipient factors (MELD), anatomical factors (biliary variations), and operative factors (transfusion, operative time, and methods of reconstruction and division of the bile duct). In particular, biliary variation with multiple bile ducts consistently emerged as a key risk factor for both donor and recipient morbidity across different cohorts. Therefore, these factors – particularly the donor’s biliary anatomy – must be thoroughly evaluated during preoperative assessments to ensure recipient safety^[47]^.

This systematic review had several limitations. First, the number of included studies and the sample sizes were limited. Only eight studies^[20–27]^ reported donor or recipient risk factors using multivariate analysis^[20–27]^, with only two being multicenter studies with adequate sample sizes^[23,25]^. Additionally, some studies included partially duplicate data^[21,22,24,26,27]^. Therefore, future multicenter studies with larger sample sizes are required for comprehensive risk factor analysis in MIDH cohorts. Second, some studies analyzed risk factors in cohorts that included donors who underwent ODH along with LDH or RDH^[20,22,26,27]^, which limits the applicability of these findings specifically to MIDH and introduces potential heterogeneity. Third, it is important to note that anatomical variations may present technical challenges not only in MIDH but also in conventional ODH. Therefore, the risk associated with complex anatomy is not unique to minimally invasive techniques. Fourth, variability in surgeon experience and institutional resources across the included studies may have influenced clinical outcomes and should be considered when assessing the external validity of the findings. In particular, results derived from high-volume centers with specialized expertise may not be readily generalizable to lower-volume or less experienced institutions. Fifth, this study is a systematic review of risk factors, and a quantitative meta-analysis was not performed due to significant heterogeneity in study designs and definitions of morbidity. As a result, the findings are limited to qualitative synthesis, and the ability to draw definitive conclusions is restricted. Finally, this review lacks an evaluation of risk factors associated with long-term outcomes in both donors and recipients. As the currently available studies primarily report short-term outcomes, future research should focus on long-term follow-up to identify prognostic factors and comprehensively evaluate the long-term risks and benefits associated with MIDH.

## Conclusion

This systematic review identified key risk factors for morbidity in donors and recipients following MIDH. Donor complications were associated with unfavorable anatomical characteristics (short hepatic ducts, multiple hepatic ducts/arteries/veins, and large graft size) and operative factors (increased surgery duration and blood loss), while recipient risk factors included biliary variations, portal vein thrombosis, hepaticojejunostomy, prolonged operative times, massive transfusion, and high MELD scores. In both donors and recipients, robotic donor surgery showed favorable outcomes and biliary variation in the graft was a key contributor to morbidity. Therefore, comprehensive preoperative assessment, meticulous surgical planning, and refinement of surgical techniques are critical in ensuring donor and recipient safety. However, due to the limited number of studies and small sample sizes, larger multicenter investigations are required to validate these findings and improve the safety and efficacy of MIDH.

## Data Availability

The data for this systematic review were obtained from publicly available published studies, and all relevant details are provided in the manuscript.

## Research registration unique identifying number (UIN)

Name of the registry: Research Registry. Unique Identifying number or registration ID: review registry1929. Hyperlink to the registration (must be publicly accessible).
